# Species Distribution of Clinical *Acinetobacter* Isolates Revealed by Different Identification Techniques

**DOI:** 10.1371/journal.pone.0104882

**Published:** 2014-08-13

**Authors:** Jianfeng Wang, Zhi Ruan, Ye Feng, Ying Fu, Yan Jiang, Haiping Wang, Yunsong Yu

**Affiliations:** 1 Department of Infectious Diseases, Sir Run Run Shaw Hospital, College of Medicine, Zhejiang University, Hangzhou, Zhejiang, China; 2 Institute for Translational Medicine, Zhejiang University, Hangzhou, Zhejiang, China; National Center for Biotechnology Information (NCBI), United States of America

## Abstract

A total of 2582 non-duplicate clinical *Acinetobacter* spp. isolates were collected to evaluate the performance of four identification methods because it is important to identify *Acinetobacter* spp. accurately and survey the species distribution to determine the appropriate antimicrobial treatment. Phenotyping (VITEK 2 and VITEK MS) and genotyping (16S rRNA and *rpoB* gene sequencing) methods were applied for species identification, and antimicrobial susceptibility test of imipenem and meropenem was performed with a disk diffusion assay. Generally, the phenotypic identification results were quite different from the genotyping results, and their discrimination ability was unsatisfactory, whereas 16S rRNA and *rpoB* gene sequencing showed consistent typing results, with different resolution. Additionally, *A. pittii*, *A. calcoaceticus* and *A. nosocomialis*, which were phylogenetically close to *A. baumannii*, accounted for 85.5% of the non-*A. baumannii* isolates. One group, which could not be clustered with any reference strains, consisted of 11 isolates and constituted a novel *Acinetobacter* species that was entitled *genomic species 33YU*. None of the non-*A. baumannii* isolates harbored a *bla*_OXA-51_-like gene, and this gene was disrupted by IS*Aba19* in only one isolate; it continues to be appropriate as a genetic marker for *A. baumannii* identification. The resistance rate of *non-A. baumannii* isolates to imipenem and/or meropenem was only 2.6%, which was significantly lower than that of *A. baumannii*. Overall, *rpoB* gene sequencing was the most accurate identification method for *Acinetobacter* species. Except for *A. baumannii*, the most frequently isolated species from the nosocomial setting were *A. pittii*, *A. calcoaceticus* and *A. nosocomialis*.

## Introduction

*Acinetobacter* is a genus of Gram-negative bacteria that are important soil organisms. Over 30 species have been identified with validly published names [1], [2]. During recent decades, *Acinetobacter* spp., particularly *A. baumannii*, have been suggested as an important public-health concern because of multiple drug resistance. The emergence of carbapenem-resistant *A. baumannii* (CRAB) has become a major international public health concern and has been described as the sentinel event of antimicrobial resistance, leaving few therapeutic options [3]. Different *Acinetobacter* spp. might possess distinct capability in invasion and virulence [4], thus ensuring the urgency of developing an accurate identification method for *Acinetobacter* spp.

The most common phenotypic and genotypic identification methods utilized in the species determination of *Acinetobacter* spp. are biochemical systems and 16S rRNA gene sequencing. Biochemical systems such as VITEK 2 are prone to being influenced by culture conditions [5]. The major limitation of 16S rRNA gene sequencing is that it is too conserved to distinguish all of the *Acinetobacter* spp. [6]. A more effective identification method is required for clinical or laboratory application. In this case, *rpoB* sequencing and Matrix-Assisted Laser Desorption/Ionization Time of Flight Mass Spectrometry (MALDI-TOF-MS) were evaluated as two alternative methods.

The *rpoB* gene has a housekeeping role, and its size differs between species, ranging from 3411 bp (*Staphylococcus aureus*) to 4185 bp (*Neisseria meningitidis*) [7]. The variability of the *rpoB* gene sequence ensures that it is impossible to design universal primers to amplify this gene for all bacteria. As a result, *rpoB* is more suitable for typing subspecies and is frequently used as a multiple-locus sequence typing (MLST) locus for many bacterial species [8]. Species is a taxonomic unit between genus and subspecies, and whether *rpoB* sequencing is feasible for species identification is unknown. Recently, many scientists have attempted to use the *rpoB* gene to identify clinical isolates of *Acinetobacter* spp [9], [10]; however, few systematic application of the *rpoB* gene have been applied to identify clinical *Acinetobacter* spp. As a result of the incompleteness of the *rpoB* database, many reference strains have not received an accurate name, which adds confusion regarding the nomenclature in the clinical field.

Mass spectrometry is a recently developed method for clinical bacterial identification that is primarily based on ribosomal proteins [11], [12]. This method has high sensitivity, high accuracy and high resolution, leading to its wide research application in life sciences and other fields. For bacterial identification, mass spectrometry has the advantage of low cost, rapidity and ease of use. Several misidentification cases in nonfermentative Gram-negative bacilli have been reported [6].

In this study, we compared the pros and cons of the four identification methods to find a convenient approach for *Acinetobacter* spp. identification at the species level.

## Materials and Methods

### Bacterial isolates

A total of 2582 non-duplicate clinical *Acinetobacter* spp. isolates were collected from 27 provinces in China from January 2009 to September 2010 [13]. Among them, 385 isolates of *bla*_OXA-51_-like-negative *Acinetobacter* spp. were selected for further identification. A total of 24 *A. baumannii* isolates, which were confirmed by *bla*_OXA-51_-like-positive and MLST, were selected as the reference, i.e., one isolate per province and ATCC17978 [13]. The primers used for amplifying the *bla*_OXA-51_-like gene are listed in Table 1. The isolates were preliminarily identified by the VITEK 2 system (Sysmex-bioMérieux, Marcy l'Etoile, France).

**Table 1:** PCR primers used for species identification.

Target gene	Primer name	Nucleotide sequence 5′→3′	Product size (bp)
**rpoB**	*rpoB*-F1	CCTTCATGACCTGGAAYGGNTA	940
	*rpoB*-R1	TCCAGGATCTGNCCNACRTTCAT	940
	*rpoB*-F2	CATGACCTGGAACGGCTAYAAYTAYGA	1210
	*rpoB*-R2	TGGTTCAGCTTCAGCATRTACATRTA	1210
16S rRNA	16S-F1	GAGTAATGCTTAGGAATCTGC	130
	16S-R1	GGTAACCGCCCTCTTTG	130
	16S-F2	GCGGACGGGTGAGTAATG	1030
	16S-R2	GCTGGCAAATAAGGAAAA	1030
*bla*_OXA-51_-like	*bla*_OXA-51_-like -F1	TAATGCTTTGATCGGCCTTG	353
	*bla*_OXA-51_-like -R1	TGGATTGCACTTCATCTTGG	353
	*bla*_OXA-51_-like -F2	TAGTGACTGCTAATCCAAAT	670
	*bla*_OXA-51_-like -R2	AAGGGAGAACGCTACAAT	670

Note: R = A/G,Y = C/T,N = A/C/G/T.

### PCR amplification and gene sequencing

The *rpoB* sequences of 32 *Acinetobacter* spp. reference strains were obtained from the NCBI GenBank database or the *Acinetobacter* spp. genomes (Table S1). The PCR degenerate primers were designed online at the region between positions 2300 and 3300 (http://blocks.fhcrc.org/codehop.html) [7]. The primers used to amplify and sequence the *rpoB* gene are listed in Table 1. The primers of the 16S rRNA gene were designed based on 32 sequences of standard strains downloaded from the website for prokaryotic nomenclature (http://www.bacterio.net/a/acinetobacter.html) (Table S2).

The interspecies diversity was evaluated using the similarities of the *rpoB* gene sequences between *A. baumannii* ATCC 17978 and reference strains of different *Acinetobacter* species. The similarities of its trimmed sequences where our in-house designed primers were located were calculated using the identical method. To accurately evaluate the highest similarity between the closest relationship groups, the genomes of 16 *A. baumannii* strains and nine *A. nosocomialis* strains were retrieved, and the complete *rpoB* gene sequences were trimmed with the identical standard to calculate their intraspecies similarity.

The sequences of the *rpoB* and 16S rRNA genes were aligned using the multiple alignment program for amino acid or nucleotide sequences (MAFFT version 7) [14]. The Neighbor-Joining (NJ) tree was constructed with the 32 reference strains and the representative isolates, which were selected from each lineages using the MEGA 5.2 program [15]–[17].

### MALDI-TOF MS

The isolates were identified on the VITEK MS system (bioMérieux, Marcyl'Etoile, France) in accordance with the manufacturer's instructions. *E. coli* ATCC 8739 was used as a quality control.

### Susceptibility test

Susceptibility testing of imipenem and meropenem was performed by disk diffusion. *A. baumannii* ATCC 17978 and *E. coli* ATCC 25922 were used for quality control. For the disk diffusion testing, a single clone was cultured at 37°C overnight in a Mueller-Hinton (MH) agar plate, recovered and diluted to 0.5 McFarland turbidity with 0.9% NaCl (w/v) solution. The diluted suspension was distributed on an MH plate with a cotton swab. After the antimicrobial susceptibility test discs were used, the plate was incubated at 37°C for 24 h. The data interpretation was performed in accordance with the Clinical and Laboratory Standards Institute (CLSI) 2012 guideline [18]. The Zone Diameter Interpretive Criteria for imipenem and meropenem were as follows: susceptible, inhibition zone ≤14 mm; intermediate susceptible inhibition zone  = 15 to 17 mm; resistant, inhibition zone ≥18 mm.

## Results

### *rpoB* sequencing

The *rpoB* gene similarities between *A. baumannii* ATCC 17978 and the reference strains of different *Acinetobacter* species were distributed as 84.8∼95.6%, and the highest similarities of these trimmed *rpoB* gene sequences were up to 96.5% (Table 2). Because the two species of *A. baumannii* and *A. nosocomialis* showed the highest interspecies similarity, the complete sequence similarity of the *rpoB* gene between them was 95.6±0.1%, whereas the similarity of their conservative region amplified by our in-house designed PCR primers was 96.6±0.2% (Table 3). The intraspecies similarity of the *A. baumannii* and *A. nosocomialis* reference strains were all above 99%, which indicated that it was an intraspecies conservative gene and, therefore, not appropriate for strain typing. Because the *rpoB* gene has the characteristics of interspecies polymorphisms and is intraspecies conservative, it could offer high discriminative power for *Acinetobacter* spp. identification. Setting a pairwise identity of 97% as the appropriate criteria for delineating this species was thus a reasonable hypothesis.

**Table 2:** The *rpoB* gene similarity between *A. baumannii* ATCC 17978 and reference strains of different species.

reference strains	A (%)	B (%)	reference strains	C (%)	D (%)
*A.genomosp.*13	95.6	96.5	*Acinetobacter sp.*_HA	87.3	89.3
*A.nosocomialis*_RUH2624	95.4	96.3	*A.johnsonii*_SH046	87.3	87.1
*A.pittii* D499	93.7	94.0	*A.bereziniae*_LMG_1003	87.3	85.6
*A.calcoaceticus*_SH024	93.3	93.6	*A.lwoffii*_WJ10621	87.3	87.0
*A.oleivorans*_DR1	92.5	94.1	*Acinetobacter sp.*_P8-3-8	87.1	85.8
*A.calcoaceticus_*RUH2202	92.0	93.2	*A.schindleri* TG19614	86.8	86.3
*A.venetianus_*RAG_1	90.0	90.8	*Acinetobacter sp.*_WC-743	86.7	85.6
*Acinetobacter_*NBRC100985	89.7	6.3	*A.tandoii_*DSM_14970	86.7	85.8
*A.tjernbergiae_*DSM_14971	89.3	89.3	*A.lwoffii*_SH145	86.6	85.4
*Acinetobacter sp._*NCTC7422	89.2	91.2	*A.baylyi*_ADP1	86.1	84.4
*A.haemolyticus_*ATCC191914	88.9	89.2	*A.ursingii_*DSM_16037	85.5	83.5
*A.parvus_*DSM16617	88.8	88.3	*A.bouvetii_*DSM_14964	85.5	84.5
*A.junii_*SH205	88.6	89.6	*A.towneri_*DSM_14962	85.4	84.3
*A.grimontii_*CIP07470	88.5	89.4	*A.soli_*CIP_110264	85.3	84.1
*A.gerneristrain_*DSM_1496	87.5	87.7	*A.radioresistens*_DSM_6976	84.8	83.4
*A.genomosp.*11	87.3	85.4			

Note: The column A and C indicated the complete *rpoB* gene sequences. The column B and D indicated the trimmed partial *rpoB* gene sequences where our designed primers located.

**Table 3:** The *rpoB* gene variation of *A.baumannii* and *A.nosocomialis* reference strains.

	Complete CDS (%)	Trimmed sequences (%)
The intraspecies similarity of *A. baumannii* (%)	99.5±0.1	99.3±0.2
The intraspecies similarity of *A. nosocomialis* (%)	99.6±0.3	99.5±0.5
The interspecies similarity (%)	95.6±0.1	96.6±0.2

**Figure 1:**
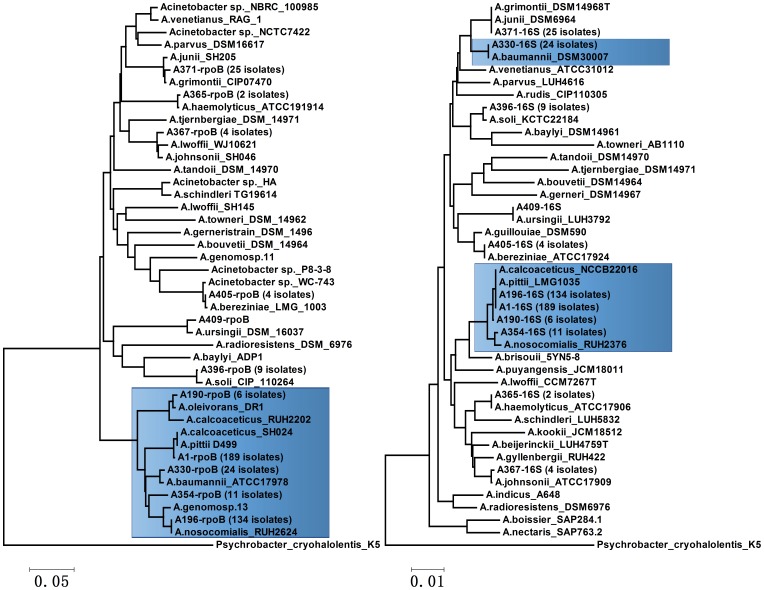
Phylogenetic relationships within different *Acinetobacter* spp. (11 representative clinical isolates, 16S rRNA gene sequences of 12 standard strains and *rpoB* gene sequences of 16 reference strains included in this study), as obtained by rooted dendrogram construction on the basis of *rpoB* gene sequences (a) and 16S rRNA gene sequences (b). The cluster analysis was performed using the MEGA 5.2 software and was based on the neighbor-joining algorithm using species of the closest related genus (i.e., *Psychrobacter cryohalolentis*) as an outgroup, with 1,000 bootstrap replications. The bar indicates 1% and 5% sequence diversity. In the phylogenetic tree of the *rpoB* gene (a), the *Acb* complex could be divided into five branches, and the evolutionary relationships among the branches were reasonable. In the phylogenetic tree of the 16S rRNA gene (b), only the *A. baumnnii* branch was separated individually from the *Acb* complex, although it was located near the branch of *A. junii*.

According to such criteria, a total of 409 clinical isolates, including 385 isolates of *bla*_OXA-51_-negative *Acinetobacter* spp. and 24 *A. baumannii* isolates, could be clearly divided into 11 lineages (Table 4, Figure 1(a)). Our study indicated that a pairwise identity of 98.5% could be set as the criterion for delineating the clinical isolates.

**Table 4:** Distribution of 409 *Acinetobacter* isolates by *rpoB* and 16S rRNA gene sequences.

Isolates No.	*rpoB*		16S rRNA	
	Classification (*n*)	Similarity (%)♦	Classification (*n*)	Similarity(%)♦
A1-A189	*A.pittii/A.calcoaceticus* (189)	99.28±0.39	*Acb* complex (189)	99.98±0.06
A190-A195	*A.oleivorans/A.calcoaceticus*_RUH2202 (6)	98.85±0.49	*Acb* complex (6)	99.82±0.13
A196-A329	*A.nosocomialis* (134)	99.00±0.33	*Acb* complex (134)	99.97±0.09
A354-A364	*A. genomic species 33YU* (11)	96.24±0.10*	*Acb* complex (11)	99.97±0.05*
A330-A353	*A.baumannii* (24)	99.17±0.07	*A.baumannii* (24)	99.69±0.11
A365-A366	*A.haemolyticus* (2)	98.75±0.24	*A.haemolyticus* (2)	99.83
A367-A370	*A.johnsonii/A.lwoffii_*WJ10621 (4)	98.53±0.32	*A.johnsonii* (4)	99.81±0.05
A371-A395	*A.junii/A.grimontii* (25)	99.61±0.34	*A.junii/A.grimontii* (25)	99.99±0.02
A396-A404	*A.soli* (9)	99.40±0.13	*A.soli* (9)	99.96±0.07
A405-A408	*A.bereziniae* (4)	99.51±0.30	*A.bereziniae* (4)	99.87±0.09
A409	*A.ursingii* (1)	98.54	*A.ursingii* (1)	99.91

Note:

* indicates the *rpoB* and 16S rRNA gene reference strain was *A.nosocomialis*_RUH2624.

♦ indicates the nucleotide similarity with reference strains.

In addition, *A. pittii* and *A. calcoaceticus* were not differentiated from each other, and their 189 isolates constituted the largest branch. The second largest branch was *A. nosocomialis*, which was composed of 134 isolates. The branch of *A. junii*/*A. grimontii* contained 25 isolates. *A. lwoffii* SH145 and *A. lwoffii* WJ10621 were located on different branches, the latter being clustered with *A. johnsonii* SH046. Similarly, *A. calcoaceticus* RUH2202 was separated from other *A. calcoaceticus* reference strains and were clustered with *A. oleivorans* isolates. Initially, *A. lwoffii* WJ10621 and *A. calcoaceticus* RUH2202 were identified incorrectly.

*A. haemolyticus, A. soli, A. bereziniae* and *A. ursingii* formed their own branch, although with a small number of isolates. One branch contained 11 isolates and did not contain any reference strains. This branch might represent a novel species, which was entitled *genomic species 33YU* (GenBank accession numbers: KF982810-KF982820).

### 16S RNA gene sequencing

A pairwise identity of 99% was set as the criterion for delineating the species of *Acinetobacter* spp. The 409 isolates were grouped into seven branches. The Neighbor-Joining (NJ) tree, which was constructed by the 32 reference strains and the representative isolates, is shown in Figure 1(a). The nucleotide similarity values of each lineage with the reference strains are list in Table 4.

*A. calcoaceticus* and *A. nosocomialis* were not differentiated by 16S rRNA gene sequencing, and these 340 isolates were combined into one large branch that was labeled *A. calcoaceticus-A. baumannii* complex (*Acb*) complex, including 11 isolates of the *genomic species 33YU* branch that were revealed by *rpoB* gene sequencing. The *A. baumannii* branch contained 24 isolates and was located near the *A. junii* branch, which was consistent with the phylogenetic relationships in other studies [19]. *A. junii* and *A. grimontii* were not differentiated from one another according to the 16S rRNA gene sequencing. The results of *A. bereziniae* and *A. soli, A. haemolyticus* and *A. ursingii* identification were identical to those by *rpoB* sequencing.

### VITEK 2 and MALDI-TOF MS

A total of 409 isolates of *Acinetobacter* spp. were divided into only four categories by the VITEK 2 system, and the result was quite different from those obtained using the other methods (Table 5). The result of VITEK MS was better than that of VITEK 2. VITEK MS showed an identical result as *rpoB* sequencing in identifying *A. junii, A. haemolyticus* and *A. johnsonii*. Eight isolates were identified as *non-Acinetobacter*, and 370 isolates were identified as the Acb complex. This result suggested that the discrimination ability of MS was unsatisfactory for *Acinetobacter* spp. identification, which could likely be resolved by further combination with *rpoB* as the reference.

**Table 5:** The difference identification results of 409 *Acinetobacter* isolates by three different methods.

Isolates No.	*rpoB* (*n*)	VITEK2 (*n*)	MALDI-TOF MS (*n*)
A1-A189	*A.pittii/A.calcoaceticus* (189)	*Acb* complex (180), *A.haemolyticus* (1)*, *A.lwoffii* (8)*	*Acb* complex (188), *E.coli* (1)*
A190-A195	*A.oleivorans/A.calcoaceticus*_RUH2202 (6)	*Acb* complex (6)	*Acb* complex (6)
A196-A329	*A.nosocomialis* (134)	*Acb* complex (131), *A.lwoffii* (3)*	*Acb* complex (134)
A354-A364	*A. genomic species 33YU* (11)	*Acb* complex (11)*	*Acb* complex (11)
A330-A353	*A.baumannii* (24)	*Acb* complex (24)	*Acb* complex (23), Non-*Acinetobater* spp. (1)*
A365-A366	*A.haemolyticus* (2)	*Acb* complex (2)*	*A.haemolyticus* (2)
A367-A370	*A.johnsonii/A.lwoffii*_WJ10621 (4)	*A.lwoffii* (2)*, *Acb* complex (2)*	*A.johnsonii* (4)
A371-A395	*A.junii/A.grimontii* (25)	*A.junii* (16), *A.haemolyticus* (2)*, *A.lwoffii* (3)*, *Acb* complex (4)*	*A.junii* (25)
A396-A404	*A.soli* (9)	*A.lwoffii* (1)*, *Acb* complex (8)*	Non-*Acinetobater* spp.(6), *Acb* complex (3)*
A405-A408	*A.bereziniae* (4)	*A.lwoffii* (4)*	*Acb* complex (4)*
A409	*A.ursingii* (1)	*Acb* complex (1)*	*Acb* complex (1)*

Note: * indicates mis-identification.

### Comparison of different methods

Because the 16S rRNA gene was more conservative than the *rpoB* gene, the former resolution was even worse than the latter. As a result, in this study, their criteria for delineating the species of *Acinetobacter* were quite different. If the pairwise identity 98.5% was set as the identical criteria for the 16S rRNA gene, most of the strains could not be differentiated. If the pairwise identity of 99% were set as the criteria for the *rpoB* gene, however, the strains would be divided into numerous clusters. According to the current standards, the consistency between these two methods was most satisfactory.

The VITEK 2 system showed unsatisfactory performance in species identification, and the result of VITEK MS was a little better. The latter method was able to identify *A. junii, A. haemolyticus* and *A. johnsonii* accurately; however, it failed to discriminate the *Acb* complex. These results could be expected because this clinical identification system relies on the database that is built on the knowledge of the understanding of the genus *Acinetobacter* by engineers and scientists. The taxonomy of *Acinetobacter* is unclear, even in the lab, and the related findings are rarely transferred to these identification systems in a timely manner.

### Susceptibility data

The resistance rate of the non-*A. baumannii* isolates to imipenem and/or meropenem was 2.6%. Among the 10 resistant isolates, three isolates belong to the *A. calcoaceticus/A. pittii* branch, and five isolates were *A. nosocomialis*, one isolate was *A. soli*, and one isolate was *genomic species 33YU*.

### False-positive rate and false negative rate of *bla*_OXA-51_-like

None of the 385 non-*A. baumannii* isolates carried the *bla*_OXA-51_-like gene. Of the 2197 *bla*_OXA-51_-like-positive *Acinetobacter* spp. isolates, one isolate was inserted by IS*Aba19*.

## Discussion

This study provided a combined genotypic and phenotypic assessment of identification methods for clinical *Acinetobacter* spp. isolates collected from 23 provinces in China. As a classic identification method, 16S rRNA gene sequencing was highly reliable at the genus level; however, it showed poor discriminatory ability on the species level [20]. The full lengths of the 16S rRNA gene sequence of *A. pittii, A. nosocomialis, A. calcoaceticus* and *A. baumannii* were nearly identical to each other. Distinguishing these species merely by 16S rRNA gene sequencing is impossible.

The high variability of the *rpoB* gene among *Acinetobacter* spp. ensured that it is appropriate for species typing; however, designing universal primers is difficult. A conserved region within the *rpoB* gene provided a unique target for primer designing [7]. Except for the different resolution, 16S rRNA and *rpoB* gene sequencing showed a consistent typing result, which indicated that both 16S rRNA and *rpoB* were rarely involved in recombination between *Acinetobacter* spp. The evolutionary tree based on the two genes could reflect their original phylogenetic relationship.

As early as 2006, *rpoB* gene sequencing was proposed for identifying species of *Acinetobacter* [21]. In 2009, Vijay A. K. B. Gundi et al. identified 99 *Acinetobacter* clinical isolates by *rpoB* gene sequencing and confirmed that an unnamed *Acinetobacter* genomic species (gen. sp.) 3 was the second dominant species after *A. baumannii* in patients [9]. Some studies reported that the *Acb* complex could be further classified into the following four species: *A. pittii, A. nosocomialis, A. calcoaceticus* and *A. baumannii* by *rpoB* gene sequences. [22], [23]. The fragment lengths of the partial *rpoB* gene amplified by their in-house designed PCR primers were only 350 and 450 bp, and the locations of the two fragments were completely different. Some of these so-called reference sequences of the *rpoB* gene have not been verified by whole genome sequencing. The similarity of the *rpoB* reference sequences in some species were even greater than 99%, e.g., *A.calcoaceticus*_SH024 and *A.pittii*_D499, whereas *A. lwoffii* WJ10621 and *A. calcoaceticus* RUH2202 were not located on their expected phylogenetic tree branches, which was consistent with their whole genome sequencing data [24]. These phenomena reiterated the current confusion of the nomenclature of *Acinetobacter* spp., the severity of which has ensured that sequences retrieved from public databases are unreliable. Excessively short fragments of the *rpoB* gene sequence might encounter marked difficulties, particularly in large-scale clinical applications, e.g., some closely related strains remain difficult to differentiate. Most researchers were continuing to follow these primers for *Acinetobacter* species identification, whereas others were attempting to replace *rpoB* gene sequencing by other housekeeping genes, e.g., *gyrB* [22]. In our study, the fragment lengths of the partial *rpoB* gene amplified using our newly re-designed PCR primers were 940 and 1210 bp, and the location of these fragments were nearly consistent. The criteria for delineating the species of *Acinetobacter* were proposed in this study, and the classification results were relatively ideal. Additionally, our study confirmed that *A. pittii/A. calcoaceticus* was the second dominant species, after *A. baumannii*, that is isolated from patients, which was consistent with a study in European countries, whereas *A. nosocomialis* was the second dominant species in South Korea [5], [9], [22]. Additionally, our study confirmed that *genomic species 33YU* was a clinically significant species in patients and should be monitored with care.

Additionally, Toı ¨di Ade' kambi et al. compared the *rpoB* gene sequence similarity of 230 bacterial species representative of 45 genera and revealed that the interspecific diversity based on the *rpoB* gene sequence was 98.2∼100%, which could be considered a suitable supplement to DNA-DNA hybridization [25]. For *Acinetobacter* species, the interspecies similarities were 84.8∼95.6%, whereas the intraspecies similarity of the complete *rpoB gene* sequences in *A. baumannii* and *A. nosocomialis* were above 99%. We hypothesize that, considering the irreplaceable advantages of *rpoB* gene sequencing, it is a promising tool for species identification because of its appropriate interspecies polymorphisms.

Vaneechoutte M et al. proposed that *A. grimontii* is a heterotypic synonym of *A. junii* by DNA-DNA hybridization and amplified fragment length polymorphism (AFLP) [26]. However, on the website of prokaryotic nomenclature, *A. grimontii* and *A. junii* are considered two different species. We found that the identity of their *rpoB* sequences was greater than 99% and did not reach the threshold of 98.5%, and we suggest combining them into one species.

The *bla*_OXA-51_-like gene has been proposed as a marker for *A. baumannii* [27]. Previous studies proposed that detecting the *bla*_OXA-51_-like gene using multiplex PCR was not reliable for the identification of *A. baumannii* because this gene could be disrupted by IS*Aba15* or IS*Aba19*, or that the *bla*_OXA-51_-like gene could be acquired by non*-A. baumannii* by horizontal gene transfer [28], [29]. We hypothesize that the above evidence was not sufficient to negate the value of the *bla*_OXA-51_-like gene as a genetic marker of *A. baumannii* in that such events were remarkably rare. Of our 2197 *A. baumannii* isolates, one isolate was inserted by IS*Aba19*, with the band of PCR product continuing, with its length increasing from 670 bp to 2000 bp. Our previous study confirmed that 875 carbapenem-resistant *bla*_OXA-51_-like-positive *Acinetobacter* isolates could be investigated using a MLST scheme, which demonstrated that they belong to *A. baumannii* [13]. None of the 385 non-*A. baumannii* isolates carried the *bla*_OXA-51_-like gene. The false positive rate and the false negative rate were low using the *bla*_OXA-51_-like gene as a genetic marker of *A. baumannii*.

The large number of our collected isolates guarantees the inclusion of most of the nosocomial *Acinetobacter* spp. One novel species, i.e., *genomic species 33YU*, was newly identified, indicating that most nosocomial *Acinetobacter* spp. have been discovered.

We suggest that *rpoB* sequencing is the best reference method for identifying *Acinetobacter* species. We designed primers for the *rpoB* gene that applied to most non-*A. baumannii* isolates successfully. A database of *rpoB* sequences was constructed, which can be used by scientists and physicians worldwide for identifying *Acinetobacter* spp. Other than *A. baumannii*, the most commonly isolated *Acinetobacter* spp. in a nosocomial environment were *A. calcoaceticus, A. pittii* and *A. nosocomialis*. Few non-*A. baumannii* showed high resistance to carbapenem, and they could be used as a control for studies to determine the reasons that *A. baumannii* evolved to be a multi-drug resistant superbug.

## Supporting Information

Table S1 The *rpoB* gene reference strains of *Acinetobacter* used in this study.
(DOCX)

Table S2 The 16S rRNA reference strains of *Acinetobacter* used in this study.
(DOCX)

## References

[1] Sieniawski K, Kaczka K, Rucinska M, Gagis L, Pomorski L (2013) Acinetobacter baumannii nosocomial infections. Pol Przegl Chir 85: 483–490. 24133105 10.2478/pjs-2013-0075

[2] Li Y, Piao CG, Ma YC, He W, Wang HM, et al (2013) Acinetobacter puyangensis sp. nov., isolated from the healthy and diseased part of Populus xeuramericana canker bark. Int J Syst Evol Microbiol 63: 2963–2969. 23396716 10.1099/ijs.0.047274-0

[3] Kaase M, Szabados F, Pfennigwerth N, Anders A, Geis G, et al.. (2013) Description of the metallo-beta-lactamase GIM-1 in Acinetobacter pittii. J Antimicrob Chemother. 10.1093/jac/dkt325 23956376

[4] Bitrian M, Solari CM, Gonzalez RH, Nudel CB (2012) Identification of virulence markers in clinically relevant strains of Acinetobacter genospecies. Int Microbiol 15: 79–88. 22847269 10.2436/20.1501.01.161

[5] Karah N, Haldorsen B, Hegstad K, Simonsen GS, Sundsfjord A, et al (2011) Species identification and molecular characterization of Acinetobacter spp. blood culture isolates from Norway. J Antimicrob Chemother 66: 738–744. 21393175 10.1093/jac/dkq521

[6] Alvarez-Buylla A, Culebras E, Picazo JJ (2012) Identification of Acinetobacter species: is Bruker biotyper MALDI-TOF mass spectrometry a good alternative to molecular techniques? Infect Genet Evol 12: 345–349. 22266021 10.1016/j.meegid.2012.01.002

[7] Adekambi T, Drancourt M, Raoult D (2009) The rpoB gene as a tool for clinical microbiologists. Trends Microbiol 17: 37–45. 19081723 10.1016/j.tim.2008.09.008

[8] Bartual SG, Seifert H, Hippler C, Luzon MA, Wisplinghoff H, et al (2005) Development of a multilocus sequence typing scheme for characterization of clinical isolates of Acinetobacter baumannii. J Clin Microbiol 43: 4382–4390. 16145081 10.1128/JCM.43.9.4382-4390.2005 PMC1234098

[9] Gundi VA, Dijkshoorn L, Burignat S, Raoult D, La Scola B (2009) Validation of partial rpoB gene sequence analysis for the identification of clinically important and emerging Acinetobacter species. Microbiology 155: 2333–2341. 19389786 10.1099/mic.0.026054-0

[10] Lee MJ, Jang SJ, Li XM, Park G, Kook JK, et al.. (2013) Comparison of rpoB gene sequencing, 16S rRNA gene sequencing, gyrB multiplex PCR, and the VITEK2 system for identification of Acinetobacter clinical isolates. Diagn Microbiol Infect Dis. 10.1016/j.diagmicrobio.2013.07.013 24157058

[11] Sedo O, Nemec A, Krizova L, Kacalova M, Zdrahal Z (2013) Improvement of MALDI-TOF MS profiling for the differentiation of species within the Acinetobacter calcoaceticus-Acinetobacter baumannii complex. Syst Appl Microbiol. 10.1016/j.syapm.2013.08.001 24054697

[12] Patel R (2013) Matrix-assisted laser desorption ionization-time of flight mass spectrometry in clinical microbiology. Clin Infect Dis 57: 564–572. 23595835 10.1093/cid/cit247

[13] Ruan Z, Chen Y, Jiang Y, Zhou H, Zhou Z, et al (2013) Wide distribution of CC92 carbapenem-resistant and OXA-23-producing Acinetobacter baumannii in multiple provinces of China. Int J Antimicrob Agents 42: 322–328. 23988720 10.1016/j.ijantimicag.2013.06.019

[14] Katoh K, Standley DM (2014) MAFFT: Iterative Refinement and Additional Methods. Methods Mol Biol 1079: 131–146. 24170399 10.1007/978-1-62703-646-7_8

[15] Saitou N, Nei M (1987) The neighbor-joining method: a new method for reconstructing phylogenetic trees. Mol Biol Evol 4: 406–425. 3447015 10.1093/oxfordjournals.molbev.a040454

[16] Kiyota N, Kushibuchi I, Kobayashi M, Tsukagoshi H, Ryo A, et al (2013) Genetic analysis of the VP4/VP2 coding region in human rhinovirus species C in patients with acute respiratory infection in Japan. J Med Microbiol 62: 610–617. 23329324 10.1099/jmm.0.049072-0

[17] Hall BG (2013) Building phylogenetic trees from molecular data with MEGA. Mol Biol Evol 30: 1229–1235. 23486614 10.1093/molbev/mst012

[18] Cockerill F (2012) Performance Standards for Antimicrobial Susceptibility Testing: Twenty-second Informational Supplement. Clinical and Laboratory Standards Institute.

[19] Gerischer U (2008) Acinetobacter molecular biology: Horizon Scientific Press.

[20] Janda JM, Abbott SL (2007) 16S rRNA gene sequencing for bacterial identification in the diagnostic laboratory: pluses, perils, and pitfalls. J Clin Microbiol 45: 2761–2764. 17626177 10.1128/JCM.01228-07 PMC2045242

[21] La Scola B, Gundi VA, Khamis A, Raoult D (2006) Sequencing of the rpoB gene and flanking spacers for molecular identification of Acinetobacter species. J Clin Microbiol 44: 827–832. 16517861 10.1128/JCM.44.3.827-832.2006 PMC1393131

[22] Lee MJ, Jang SJ, Li XM, Park G, Kook JK, et al (2014) Comparison of rpoB gene sequencing, 16S rRNA gene sequencing, gyrB multiplex PCR, and the VITEK2 system for identification of Acinetobacter clinical isolates. Diagn Microbiol Infect Dis 78: 29–34. 24157058 10.1016/j.diagmicrobio.2013.07.013

[23] Nemec A, Krizova L, Maixnerova M, van der Reijden TJ, Deschaght P, et al (2011) Genotypic and phenotypic characterization of the Acinetobacter calcoaceticus-Acinetobacter baumannii complex with the proposal of Acinetobacter pittii sp. nov. (formerly Acinetobacter genomic species 3) and Acinetobacter nosocomialis sp. nov. (formerly Acinetobacter genomic species 13TU). Res Microbiol 162: 393–404. 21320596 10.1016/j.resmic.2011.02.006

[24] Sahl JW, Gillece JD, Schupp JM, Waddell VG, Driebe EM, et al (2013) Evolution of a pathogen: a comparative genomics analysis identifies a genetic pathway to pathogenesis in acinetobacter. PLoS One 8: e54287. 23365658 10.1371/journal.pone.0054287 PMC3554770

[25] Adekambi T, Shinnick TM, Raoult D, Drancourt M (2008) Complete rpoB gene sequencing as a suitable supplement to DNA-DNA hybridization for bacterial species and genus delineation. Int J Syst Evol Microbiol 58: 1807–1814. 18676461 10.1099/ijs.0.65440-0

[26] Vaneechoutte M, De Baere T, Nemec A, Musilek M, van der Reijden TJ, et al (2008) Reclassification of Acinetobacter grimontii Carr, et al. 2003 as a later synonym of Acinetobacter junii Bouvet and Grimont 1986. Int J Syst Evol Microbiol 58: 937–940. 18398198 10.1099/ijs.0.65129-0

[27] Turton JF, Woodford N, Glover J, Yarde S, Kaufmann ME, et al (2006) Identification of Acinetobacter baumannii by detection of the blaOXA-51-like carbapenemase gene intrinsic to this species. J Clin Microbiol 44: 2974–2976. 16891520 10.1128/JCM.01021-06 PMC1594603

[28] Zander E, Higgins PG, Fernandez-Gonzalez A, Seifert H (2013) Detection of intrinsic blaOXA-51-like by multiplex PCR on its own is not reliable for the identification of Acinetobacter baumannii. Int J Med Microbiol 303: 88–89. 23375845 10.1016/j.ijmm.2012.12.007

[29] Lee YT, Kuo SC, Chiang MC, Yang SP, Chen CP, et al (2012) Emergence of carbapenem-resistant non-baumannii species of Acinetobacter harboring a blaOXA-51-like gene that is intrinsic to A. baumannii. Antimicrob Agents Chemother 56: 1124–1127. 22083478 10.1128/AAC.00622-11 PMC3264228

