# Supplementary material for: Species Distribution of Clinical Acinetobacter Isolates Revealed by Different Identification Techniques
Source: PLoS One. 2014 Aug 13;9(8):e104882. doi: 10.1371/journal.pone.0104882 (PMC4132069; doi:10.1371/journal.pone.0104882)
Supplement: Table S1 — The rpoB gene reference strains of Acinetobacter used in this study. (DOCX) [file pone.0104882.s001.docx]

**Table S1 The *rpoB* gene reference strains of *Acinetobacter* used in this study**

| ***No.*** | ***Acinetobacter species*** | ***GenBank accession no.*** | ***GC content*** |
| --- | --- | --- | --- |
| 1 | *A.baumannii*_ATCC17978 | NC_009085 | 41.74% |
| 2 | *Acinetobactersp.*RUH2624 | NZ_ACQF00000000 | 41.59% |
| 3 | *A.calcoaceticus_*SH024 | NZ_ADCH01000000 | 41.72% |
| 4 | *A.calcoaceticus_*RUH2202 | NZ_ACPK01000000 | 41.72% |
| 5 | *Acinetobactersp._*NBRC_100985 | NZ_BAEB01000000 | 42.09% |
| 6 | *A.parvus_*DSM16617 | NZ_AIEB01000000 | 43.24% |
| 7 | *A.haemolyticus*_ATCC191914 | NZ_ADMT01000000 | 43.24% |
| 8 | *A.junii_*SH205 | NZ_ACPM01000000 | 42.04% |
| 9 | *Acinetobactersp._*HA | NZ_AJXD01000000 | 44.74% |
| 10 | *A.lwoffii_*WJ10621 | NZ_AFQY01000000 | 43.76% |
| 11 | *A.johnsonii_*SH046 | NZ_ACPL01000000 | 43.64% |
| 12 | *A.ursingii_*DSM_16037 | NZ_AIEA01000000 | 43.59% |
| 13 | *A.lwoffii_*SH145 | NZ_ACPN01000000 | 44.76% |
| 14 | *A.bereziniae_*LMG_1003 | NZ_AIEI01000000 | 42.22% |
| 15 | *Acinetobactersp._*WC-743 | NZ_AMFQ01000000 | 42.12% |
| 16 | *Acinetobactersp._*P8-3-8 | NZ_AFIE01000000 | 41.37% |
| 17 | *A.radioresistens_*DSM_6976 | NZ_BAGY01000000 | 45.41% |
| 18 | *Acinetobactersp._*NCTC7422 | NZ_AIED01000000 | 43.88% |
| 19 | *A.oleivorans_*DR1 | NC_014259 | 41.18% |
| 20 | *Acinetobacter* baylyi | NC_005966 | 44.19% |
| 21 | *A.bouvetii_*DSM_14964 | NZ_AREL01000000 | 46.12% |
| 22 | *A.genomosp.*11 | NZ_KB849456 | 41.92% |
| 23 | *A.gerneristrain_*DSM_14967 | NC_APPN01000000 | 41.38% |
| 24 | *A.tandoii_*DSM_14970 | NZ_AQFM01000000 | 43.34% |
| 25 | *A.tjernbergiae_*DSM_14971 | NZ_ARFU00000000 | 42.31% |
| 26 | *A.towneri_*DSM_14962 | NZ_APPY01000000 | 44.71% |
| 27 | *A.venetianus_*RAG_1 | NZ_AKIQ01000000 | 42.30% |
| 28 | *A.genomosp.13_*NCTC8102 | NZ_AIEJ01000000 | 41.67% |
| 29 | *A.pittii_*D499 | NZ_AGFH01000000 | 41.87% |
| 30 | *A.schindleri_*TG19614 | NZ_AMJK01000000 | 44.75% |
| 31 | *A.soli_*CIP_110264 | NC_APPU01000000 | 46.42% |
| 32 | *A.grimontii_*CIP07470 | DQ207483 | 41.97% |
| 33 | *A.baumanii_*NCTC10304 | NZ_AIEE01000087 | 41.77% |
| 34 | *A.baumanii_*307-0294 | NC_011595 | 41.70% |
| 35 | *A.baumanii_*1656-2 | NC_017162 | 41.63% |
| 36 | *A.baumanii_*MDR-TJ | NC_017847 | 41.67% |
| 37 | *A.baumanii_*MDR-ZJ06 | NC_017171 | 41.67% |
| 38 | *A.baumanii_*TYTH-1 | NC_018706 | 41.67% |
| 39 | *A.baumannii_*ACICU_uid58765 | NC_010605 | 41.69% |
| 40 | *A.baumannii_*AB0057 | NC_011586 | 41.82% |
| 41. | *A.baumannii_*AYE_uid61637 | NC_010410 | 41.82% |
| 42 | *A.baumannii_*BJAB0715 | NC_021733 | 41.82% |
| 43 | *A.baumannii_*BJAB0868 | NC_021729 | 41.69% |
| 44 | *A.baumannii_*BJAB07104 | NC_021726 | 41.69% |
| 45 | *A.baumannii_*D1279779 | NC_020547 | 41.77% |
| 46 | *A.baumannii_*SDF | NC_010400 | 41.67% |
| 47 | *A.baumannii_*TCDC-AB0715 | NC_017387 | 41.69% |
| 48 | *A.baumannii_*ZW85 | NC_017387 | 41.77% |
| 49 | *A.genomosp.*13TU | NZ_AIEJ01000035 | 41.69% |
| 50 | *A.nosocomialis_*28F | NZ_CBSD020000001 | 41.57% |
| 51 | *A.nosocomialis_*M2c_7 | NZ_AWOW01000009 | 41.74% |
| 52 | *A.nosocomialis_*NIPH-386 | NZ_APPP01000003 | 41.59% |
| 53 | *A.nosocomialis_*NIPH2119 | NZ_APOP01000014 | 41.69% |
| 54 | *A.nosocomialis_*TG19596 | NZ_AMIZ01000046 | 40.99% |
| 55 | *A.nosocomialis_*TG21145 | NZ_AMJH01000014 | 41.69% |
