# Supplementary material for: Species Distribution of Clinical Acinetobacter Isolates Revealed by Different Identification Techniques
Source: PLoS One. 2014 Aug 13;9(8):e104882. doi: 10.1371/journal.pone.0104882 (PMC4132069; doi:10.1371/journal.pone.0104882)
Supplement: Table S2 — The 16S rRNA reference strains of Acinetobacter used in this study. (DOCX) [file pone.0104882.s002.docx]

**Table S2 The 16S rRNA reference strains of *Acinetobacter* used in this study**

| **Acinetobacter species** | **reference strain** | **16SrRNA GenBank accession no.** | **References** |
| --- | --- | --- | --- |
| A.baumannii | DSM30007 | X81660 | Int. J. Syst. Bacteriol, 1986, 36, 228-240. |
| A.baylyi | DSM 14961 | AF509820 | Int. J. Syst. Evol. Microbiol., 2003, 53, 953-963 |
| A.beijerinckii | LUH 4759T | AJ626712 | Int. J. Syst. Evol. Microbiol., 2009, 59, 118-124 |
| A.bereziniae | ATCC 17924 | Z93443 | Int. J. Syst. Evol. Microbiol., 2010, 60, 896-903. |
| A.boissieri | SAP 284.1 | [JQ771141](http://www.ncbi.nlm.nih.gov/sites/entrez?term=JQ771141&cmd=Search&db=nuccore) | Int. J. Syst. Evol. Microbiol., 2013, 63,1532-1539 |
| A.bouvetii | DSM 14964 | [AF509827](http://www.ncbi.nlm.nih.gov/sites/entrez?term=AF509827&cmd=Search&db=nuccore) | Int. J. Syst. Evol. Microbiol., 2003, 53, 953-963 |
| A.brisouii | 5YN5-8 | [DQ832256](http://www.ncbi.nlm.nih.gov/sites/entrez?term=DQ832256&cmd=Search&db=nuccore) | J. Microbiol., 2010, 48, 36-39 |
| A.calcoaceticus | NCCB 22016 | [AJ888983](http://www.ncbi.nlm.nih.gov/sites/entrez?term=AJ888983&cmd=Search&db=nuccore) | Int. J. Syst. Bacteriol., 1980, 30, 225-420 |
| A.gerneri | DSM 14967 | [AF509829](http://www.ncbi.nlm.nih.gov/sites/entrez?term=AF509829&cmd=Search&db=nuccore) | Int. J. Syst. Evol. Microbiol., 2003, 53, 953-963 |
| A.guillouiae | DSM590 | [X81659](http://www.ncbi.nlm.nih.gov/sites/entrez?term=X81659&cmd=Search&db=nuccore) | Int. J. Syst. Evol. Microbiol., 2010, 60, 896-903 |
| A.grimontii | DSM 14968 T | [EF611411](http://www.ncbi.nlm.nih.gov/sites/entrez?term=EF611411&cmd=Search&db=nuccore) | Int. J. Syst. Evol. Microbiol., 2003, 53, 953-963 |
| A. gyllenbergii | RUH 422 | [AJ293694](http://www.ncbi.nlm.nih.gov/sites/entrez?term=AJ293694&cmd=Search&db=nuccore) | Int. J. Syst. Evol. Microbiol., 2009, 59, 118-124 |
| A.haemolyticus | ATCC 17906 | [X81662](http://www.ncbi.nlm.nih.gov/sites/entrez?term=X81662&cmd=Search&db=nuccore) | Int. J. Syst. Bacteriol., 1986, 36, 228-240 |
| A.indicus | A648 | [HM047743](http://www.ncbi.nlm.nih.gov/sites/entrez?term=HM047743&cmd=Search&db=nuccore) | Int. J. Syst. Evol. Microbiol., 2012, 62, 2883-2890 |
| A.johnsonii | ATCC 17909 | [Z93440](http://www.ncbi.nlm.nih.gov/sites/entrez?term=Z93440&cmd=Search&db=nuccore) | Int. J. Syst. Bacteriol., 1986, 36, 228-240 |
| A.junii | DSM 6964 | [X81664](http://www.ncbi.nlm.nih.gov/sites/entrez?term=X81664&cmd=Search&db=nuccore) | Int. J. Syst. Bacteriol., 1986, 36, 228-240 |
| A.lwoffii | CCM 7267T | DSM2403 | Int. J. Syst. Bacteriol., 1980, 30, 225-420 |
| A.nectaris | SAP763.2 | [JQ771132](http://www.ncbi.nlm.nih.gov/sites/entrez?term=JQ771132&cmd=Search&db=nuccore) | Int. J. Syst. Evol. Microbiol.,??? |
| A.nosocomialis | RUH2376 | [HQ180192](http://www.ncbi.nlm.nih.gov/sites/entrez?term=HQ180192&cmd=Search&db=nuccore) | Res. Microbiol., 2011, 162, 393-404 |
| A.parvus | LUH4616 | AJ293691 | Int. J. Syst. Evol. Microbiol., 2003, 53, 1563-1567 |
| A.pittii | LMG1035 | HQ180184 | Res. Microbiol., 2011, 162, 393-404 |
| A.radioresistens | DSM6976 | X81666 | Int. J. Syst. Bacteriol., 1988, 38, 209-211 |
| A.rudis | CIP110305 | EF204258 | Int. J. Syst. Evol. Microbiol., 2011, 61, 2837-2843 |
| A.shindleri | LUH 5832 | [AJ278311](http://www.ncbi.nlm.nih.gov/sites/entrez?term=AJ278311&cmd=Search&db=nuccore) | Int. J. Syst. Evol. Microbiol., 2001, 51, 1891-1899 |
| A.soli | KCTC 22184 | [EU290155](http://www.ncbi.nlm.nih.gov/sites/entrez?term=EU290155&cmd=Search&db=nuccore) | J. Microbiol., 2008, 46, 396-401 |
| A.tandoii | DSM 14970 | AF509830 | Int. J. Syst. Evol. Microbiol., 2003, 53, 953-963 |
| A.tjernbergiae | DSM 14971 | AF509825 | Int. J. Syst. Evol. Microbiol., 2003, 53, 953-963 |
| A.towneri | AB1110 | [AF509823](http://www.ncbi.nlm.nih.gov/sites/entrez?term=AF509823&cmd=Search&db=nuccore) | Int. J. Syst. Evol. Microbiol., 2003, 53, 953-963 |
| A.ursingii | LUH 3792 | [AJ275038](http://www.ncbi.nlm.nih.gov/sites/entrez?term=AJ275038&cmd=Search&db=nuccore) | Int. J. Syst. Evol. Microbiol., 2001, 51, 1891-1899 |
| A.venetianus | ATCC 31012 | [AJ295007](http://www.ncbi.nlm.nih.gov/sites/entrez?term=AJ295007&cmd=Search&db=nuccore) | Res. Microbiol,1997, 148, 237-249 |
| A.kookii | JCM 18512 | [JX137279](http://www.ncbi.nlm.nih.gov/sites/entrez?term=JX137279&cmd=Search&db=nuccore) | [Int J Syst Evol Microbiol.](http://www.ncbi.nlm.nih.gov/pubmed/?term=Int.+J.+Syst.+Evol.+Microbiol.%2C+63%2C+4402-4406.) 2013 Dec;63(Pt 12):4402-6 |
| A.puyangensis | JCM 18011 | [JN664255](http://www.ncbi.nlm.nih.gov/sites/entrez?term=JN664255&cmd=Search&db=nuccore) | [Int J Syst Evol Microbiol.](http://www.ncbi.nlm.nih.gov/pubmed/?term=Int.+J.+Syst.+Evol.+Microbiol.%2C+63%2C+2963-2969.) 2013 Aug;63(Pt 8):2963-9 |
